# Selection of Sperm Based on Hypo-Osmotic Swelling May Improve ICSI Outcome: A Preliminary Prospective Clinical Trial

**Published:** 2014-03-09

**Authors:** Nasim Charehjooy, Mohammad Hassan Najafi, Marziyeh Tavalaee, Mohammad Reza Deemeh, Leila Azadi, Abdol Hossein Shiravi, Mohammad Hossein Nasr-Esfahani

**Affiliations:** 1 Department of Reproductive Biotechnology at Reproductive Biomedicine Research Center, Royan Institute for Biotechnology, ACECR, Isfahan, Iran; 2 Department of Biology, Damghan Branch, Islamic Azad University, Damghan, Iran; 3 Isfahan Fertility and Infertility Center, Isfahan, Iran

**Keywords:** HOST, ICSI, Fertilization, Implantation, Pregnancy

## Abstract

**Background:**

The intra-cytoplasmic sperm injection (ICSI) technique selects sperm according to morphology and motility. However, these parameters cannot predict the chromatin integrity of sperm. Considering the detrimental effects of DNA-damaged sperm on reproductive outcomes, novel sperm selection procedures have been proposed to circumvent the possibility of inseminating DNA-damaged sperm. It has been shown that different potential hypo-osmotic swelling test (HOST) patterns possess the potential to differentiate between sperm that have intact or damaged chromatin. Therefore, for the first time, this preliminary study evaluates the role of HOST as a sperm selection procedure in a clinical setting.

**Materials and Methods:**

In this preliminary prospective clinical trial study, we divided infertile couples diagnosed with male infertility into two groups. In the treatment group (n=39), half of the oocytes were inseminated by sperm selected following density gradient centrifugation (DGC group). The remaining oocytes from the treatment group were inseminated by sperm chosen according to HOST pattern (c, d or e) following DGC processing (HOST group). In the control group (n=63), all oocytes were inseminated by sperm chosen after DGC.

**Results:**

There was a significantly higher percentage of embryos that had good quality, implantation, and chemical pregnancy rates in the HOST group compared to the DGC group (p≤0.05).

**Conclusion:**

: This study has shown that selecting sperm according to membrane functionality (HOST pattern) rather morphology and viability may open a new window in our approach for determining the appropriate sperm for ICSI, particularly in individuals with severe male infertility (Registration Number: IRCT201307087223N2).

## Introduction

Although intra-cytoplasmic sperm injection (ICSI) has revolutionized the treatment of male infertility, its safety is still debatable. During ICSI, motile sperm with normal morphology are visually selected for insemination of an oocyte (1). A study by Avendaño and Oehninger in 2011 has shown that the percentage of sperm with normal morphology, presenting DNA fragmentation increases in individuals with severe male infertility (2). 

Because the ICSI procedure bypasses natural sperm selection barriers and increases the chance of insemination of a defective or DNA damaged sperm in severe male infertility cases, it is believed that insemination of these sperm should be avoided. The consequences of insemination of damaged sperm not only result in reduced ICSI outcomes in terms of fertilization, blastocyst formation, implantation, pregnancy and live birth rates, however there may be other consequences such as an increased cancer rate after birth that have yet to be determined (3-5).

To avoid insemination of defective sperm, researchers in the field of sperm biology have focused on novel sperm selection procedures (for more detail see Nasr-Esfahani et al. and Said and Land) (6, 7). Among these novel approaches is the ability of sperm to respond to hypoosmotic stress. The hypo-osmotic swelling test (HOST) is considered to have this potential (8).

HOST was initially introduced as a functional assay for infertility diagnosis by Jeyendran et al. (9). In some spermatozoa the plasma membrane may be physically intact (live sperm), but functionally inactive. Therefore HOST provides supplementary information about sperm function. Rossato et al. have demonstrated that sperm exposure to hypo-osmotic conditions leads to water influx into the cytoplasm, an expanding sperm volume which stretches the sperm membrane, resulting in opening osmosensitive calcium channels and calcium influx within the sperm’s cytoplasm following induction of an acrosome reaction (10, 11). Expansion of sperm volume leads to different tail swelling patterns which are classified by WHO as a-sperm to g-sperm (12).

Pattern a or a-sperm have no expansion and are considered nonviable and nonfunctional. In patterns b-sperm to g-sperm, volume expansion indicates different degrees of integrity and functionality of the sperm’s membrane.

Recently, Stanger et al. have assessed DNA integrity of different sperm patterns and showed that d-, e-, and f-sperm are associated with minimal DNA damage (13). Further detailed evaluation by Bassiri et al. have also revealed that b-, c-, and d-sperm are associated with a minimal percentage and frequency of DNA damage, protamine deficiency, abnormal morphology and apoptosis. The best sperm are considered to be from the c- and d-sperm (8). According to both studies, a- and g-sperm are associated with the most anomalies, especially DNA damage. Thus insemination of g-sperm as well as nonviable a-sperm should be avoided during ICSI. These results possibly show that the degree of sperm volume expansion may be related to quality and particularly sperm functionality. The best sperm are d-sperm that have moderate expand- ing capacity, which is in accordance with the observed results in both studies (8, 13). According to this hypothesis, the next best sperm are c- and e-sperm according to Bassiri et al. (14) who chose c-sperm, whereas Stanger et al. chose esperm as the next best sperm (13). Analysis of correlation between sperm DNA fragmentation in sperm with different HOST patterns also support the above hypothesis.

The aim of this study was to evaluate, for the first time, the potential of HOST in a clinical setting. In the treatment group we divided sibling oocytes into two subgroups, i. oocytes inseminated by routine ICSI procedure following density gradient centrifugation (DGC group) and ii. oocytes inseminated by sperm chosen according to their HOST pattern following DGC processing (HOST group). The control group consisted of couples undergoing routine ICSI. The clinical outcomes of this group (control) following embryo transfer were compared with the clinical outcomes of couples who received embryo transfer solely from the HOST group.

## Materials and Methods

In this preliminary prospective clinical trial study, semen samples were obtained from 102 infertile individuals who referred to the Isfahan Fertility and Infertility Center for ICSI procedures from 2011 to 2012. All couples were informed about the study and consent forms were signed by all participants. Semen samples were collected by individuals through the process of masturbation, after three or four days of abstinence, on the day of oocyte retrieval. Routine semen analysis was carried out by light microscopy according to World Health Organization (WHO) criteria (12). This study approved by Ethical Committee of Royan Institute.

### Patients

**Fig 1:**
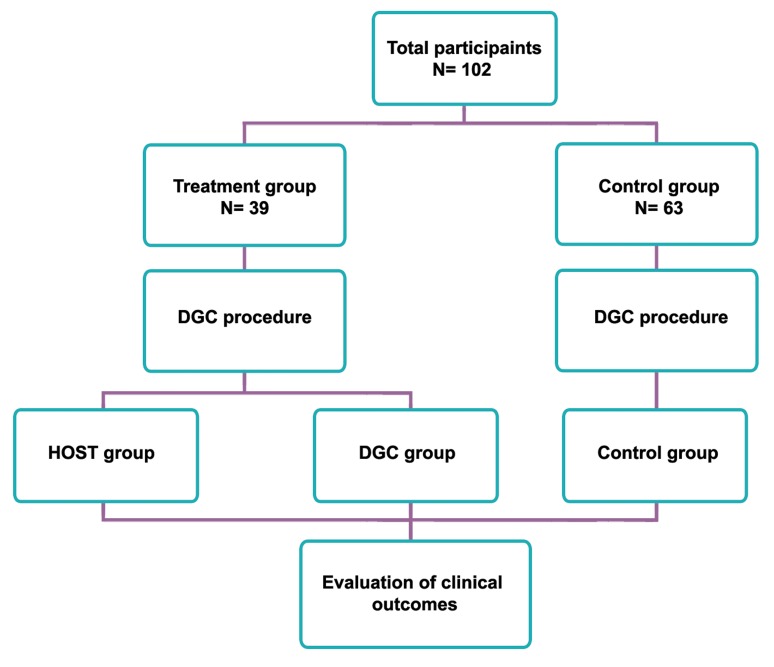
Study design. Hypo-osmotic swelling test (HOST) and Density gradient centrifugation (DGC).

**Fig 2:**
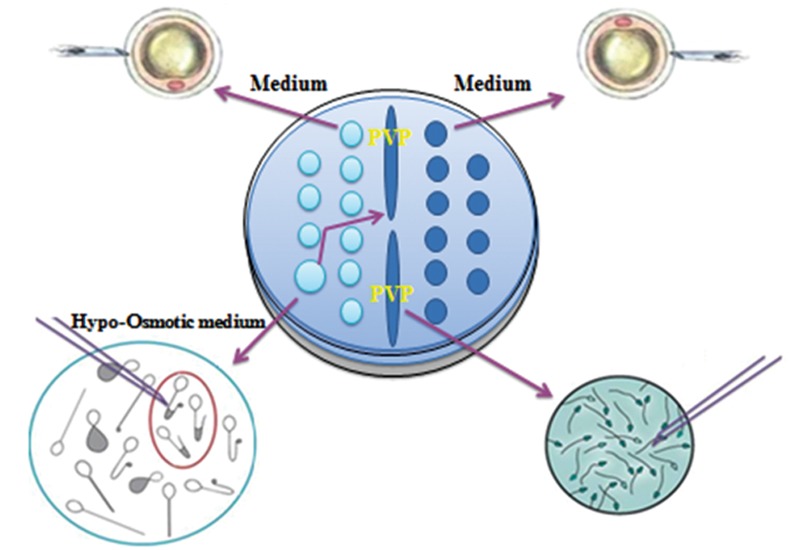
Routine density gradient centrifugation (DGC) and novel hypo-osmotic swelling test (HOST) sperm selection procedures.

Infertile couples were informed about the study and grouped according to their preference for inclusion in either the treatment or control groups (Fig 1). In the treatment group, half of the oocytes were inseminated by sperm selected following DGC (DGC group) whereas the remaining oocytes were inseminated by sperm selected according to the HOST pattern following DGC processing (HOST group). In the HOST group, sperm were initially selected according to their viability and morphology, after which they were exposed to hypotonic conditions. Subsequently, individual sperm were selected according to the HOST pattern. Once chosen by the HOST pattern (c, d and e) sperm were washed and transferred to ICSI-100 drops before insemination. In this study, sperm with a or g patterns were not inseminated. In the control group all oocytes were inseminated by sperm selected based on viability and morphology following DGC (Fig 2).

We recruited couples into the study until the treatment group reached 39 couples based on a sample size formula constructed according to a 95% confidence coefficient and 80% power of study.

Couples with primary male infertility included in this study had at least two abnormal semen parameters according to the WHO-2010. Those with less than six normal appearing, mature metaphase II oocytes were excluded. We excluded couples over the age of 40 years from the study.

### ICSI outcomes

We performed sperm processing, super ovulation, the ICSI procedure and embryo culture according to our previous study (15). The fertilization rate was assessed around 16-18 hours post-ICSI according to the presence of two pronuclei. Percentage of fertilization for each case was calculated by taking into consideration the ratio of fertilized oocytes to the total number of survived injected metaphase II (MII) oocytes, which was then multiplied by 100.

We assessed embryo quality three days post-oocyte retrieval according to Nasr-Esfahani et al. using a three-point scoring system and taking into consideration the following parameters: absence of fragmentation and/or fragmentation rate less than 25% of the embryonic surface; equality of blastomere size and shape; and the number of cells (greater or less than eight). We compared the chemical pregnancy, clinical pregnancy, implantation and abortion rates between treatment and control groups (15).

Embryologists who divided the oocytes into two groups, scored the embryos and followed the cycle outcomes were blinded to treatment assignments.

### Simultaneous assessment of DNA fragmentation and sperm morphology 

In order to evaluate the correlation between DNA fragmentation and fertilization in sibling oocytes (DGC vs. HOST group), we evaluated DNA fragmentation by terminal deoxynucleotidyl transferase mediated dUTP nick end labeling (TUNEL) in the treatment semen samples according to Tavalaee et al. (16). Of note, following sperm assessment under UV, the sperm were additionally observed with light microscopy. To the best of the technician’s ability sperm normality was assessed and the percentage of TUNEL negative sperm with normal morphology was deter- mined. The aim of this procedure was to assess the correlation between fertilization rate and percentage of TUNEL negative sperm with normal morphology in oocytes inseminated in the DGC or HOST groups. 

### Statistical analysis

The mean, range of variables, Pearson’s corre- lation coefficient, student’s t test (paired and independent sample) and chi-square test were performed using the Statistical Package for the Social Studies (SPSS 11.5; Chicago, IL, USA) software for correlation analyses and a comparison of the results between different procedures. Values are presented as mean ± SEM in the Results and tables. P<0.05 was considered to be significant. 

## Results

Descriptive analysis of sperm parameters and characteristics of couples in the treatment and control groups are presented in table 1. These parameters were similar between the two groups, except for female age which was higher in the control group.

**Table 1:** Mean semen parameters and couples’ characteristics between treatment and control groups.


Parameters	Treatment	Control

**Sperm concentration (million/ml)**	17.5 ±2.7	14.3±1.7
	0.01-67	0.2-60
**Sperm motility (%)**	21.8±2.6	19.5±1.5
	0-65	0-70
**Abnormal morphology (%)**	94.7±0.8	95.8±0.4
	82-100	80-100
**Female age (Y)**	28.7±0.8^a^	30.8±0.6^a^
	20-40	19-40
**Male age (Y)**	34±0.9	36.1±0.7
	25-50	26-51
**Duration of marriage (Y)**	6.48±0.7	7.3±0.6
	1.5-22	1-20
**Duration of infertility age (Y)**	5.6±0.6	7.3±0.6
	1-20	1-20
**Number of oocytes**	13.9±0.6	8.2±0.4
	6-27	4-18


Values are mean ± SE. Common letters represent significant difference (p < 0.05).

**Fig 3:**
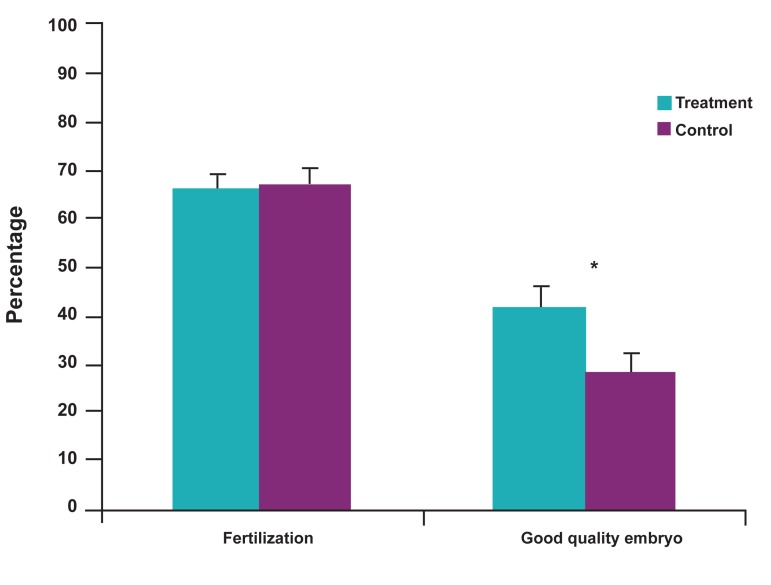
Comparison of fertilization and good embryo quality rates between treatment and control groups. Asterisk: Significant difference at p<0.05.

According to table 2, the fertilization rates were 67.9 ± 3.5 (DGC), 68.1 ± 2.9 (HOST) and 66.9 ± 2.6 (control); percentages of good quality embryos were 28.9 ± 4.2 (DGC), 42 ± 5.7 (HOST) and 31.4 ± 2.8 (control); percentage of moderate quality embryos were 40.3 ± 4.9 (DGC), 34.5 ± 5.6 (HOST) and 46.1 ± 2.8 (control); and the percentage of poor quality embryos were 30.6 ± 3.8 (DGC), 23.4 ± 4.9 (HOST) and 22.1 ± 2.8 (control). The fertilization rate in the HOST group was insignificantly higher compared to the other two groups. There were a significantly higher percentage of good quality embryos (p=0.03) in the HOST group compared to the sibling oocytes in the DGC group (Fig 3).

**Table 2:** Percentage of fertilization and embryo quality between hypo-osmotic swelling test (HOST), density gradient centrifugation (DGC) and control groups


Parameters	Treatment		Control
	DGC	HOST	

**Fertilization rate **	67.9±3.5	68.1±2.8	66.9±2.6
**Good embryo quality rate**	28.9±4.2^a^	42±5.7^a^	31.4±2.8
**Moderate embryo quality rate**	40.3±4.9	34.5±5.6^b^	46.1±2.8^b^
**Poor embryo quality rate**	30.6±3.8	23.4±4.9	22.1±2.8


Values are mean ± SE. Common letters represent significant difference (p<0.05).

Embryo selection for transfer was based on the availability of good quality embryos. Therefore out of 39 couples in the treatment group, 25 received embryos from the HOST group and the remaining 14 couples received embryos from both the DGC and/or HOST groups. We compared implantation, pregnancy, and abortion rates between treatment group couples who received embryos from the HOST procedure with the control group.

We also assessed the percentage of DNA fragmentation by TUNEL in sperm with normal or abnormal morphology. According to the results, there was a significant correlation between the percentage of TUNEL negative sperm with normal morphology and fertilization rate in DGC group (r=0.4, p=0.00), but not in the HOST group (r=0.1, p=0.4).

The chemical pregnancy rate percentages were evaluated in the treatment and control groups. Out of 25 infertile individuals in the treatment group, 12 (48%) became pregnant. In the control group 13 out of 58 (22.4%) became pregnant, which was significant (p=0.02). In addition, the clinical pregnancy rate was 34.7% (8 out of 23) for the treatment group compared to 21.0% (12 out of 57) for the control group which was insignificantly higher (Table 3). The implantation rate was 21.6% (13 out of 60) in the treatment group and 9.3% (15 out of 160) in the control group, which was significant (p=0.01). There was no significant difference in abortion rate between the treatment and control groups.

**Table 3:** Clinical outcomes between treatment and control group


Groups	Chemical pregnancy ß-hCG+/No. ET	Clinical pregnancy Heart beat/No.ET	Implantation rate No. Sac/No. ET	Abortion rate

**Treatment**	48% (12/25)	34.7% (8/23)	21.7% (13/60)	0% (0/8)
**Control**	22.4% (13/58)	21% (12/57)	9.3% (15/160)	0% (0/15)
**P value**	0.02	0.1	0.01	……


## Discussion

In conventional ICSI sperm selection is based on motility and morphology, which does not alleviate the likelihood of inseminating sperm that have subtle defects (17, 18). Therefore, selection of sperm based on other functional characteristics remains at the forefront of sperm biology research. 

Spermolemma, the sperm’s outer layer, can become damaged when exposed to non physiological conditions, toxicants such as reactive oxygen species (ROS) or by internal factors that induce apoptosis. Under these circumstances the genome integrity is also affected. Therefore, integrity of the sperm’s membrane may be taken as an index for cellular integrity (19) and sperm selection according to membrane integrity may provide a solution for choosing suitable sperm for ICSI. Currently, there are few methods that detect live sperm that have an intact membrane, including i. HOST (20), ii. sperm tail flexibility (21), and iii. single laser shot tests (22). Among these, HOST not only assesses viability but it also reflects the functionality of the sperm membrane. Based on previous study, it has been proposed that sperm with c, d and e HOST patterns have higher integrity than gpattern sperm. Therefore, it has been proposed that g-pattern sperm should not be used for ICSI (8).

In this study, we divided the oocytes from each couple into two subgroups, where one-half were inseminated by a routine sperm selection procedure and the other half underwent insemination using the HOST procedure (Fig 2). The results revealed no significant differences for fertilization rates between the sibling oocytes. In addition, the fertilization rates in sibling oocytes were similar to the control group. The percentage of good quality embryos was significantly higher in the HOST group compared to the sibling oocytes (DGC group). Thus, it appeared that embryos derived from sperm selected by HOST had higher developmental potential compared to embryos derived when conventional ICSI was implemented. This effect might be attributed to the quality of selected sperm. However, the percentage of good quality embryos (31.4%) in the control group was insignificantly lower than the HOST group (42%), despite similar semen parameters in both groups. There was a significant difference in percentages of chemical pregnancies between the HOST (48%) and control (22.4%) groups.

Implantation rates percentages also significantly differed between the HOST (21.7%) and control (9.3%) groups. The implantation rate in the control group was lower than the rates commonly reported in the literature and obtained in our center. This was likely due to the type of patients (severe male infertility) chosen for this study. Clinical pregnancies were also higher in the HOST (34.7%) compared to the control (21%) group, but this difference was insignificant.

Recently, Avendaño et al. have demonstrated that ICSI outcomes did not show a significant relationship with DNA fragmentation in whole semen samples, rather a significant correlation was observed when the degree of DNA fragmentation was evaluated in sperm that had normal morphology in each sample. Therefore, they have concluded that "the evaluation of DNA integrity in morphologically normal spermatozoa after sperm selection is a better approach to examine sperm DNA fragmentation and any potential impact on ICSI procedure" (2). Thus, we assessed the percentage of DNA fragmentation in the normal and abnormal sperm populations of each sample. We observed a significant correlation between the percentage of sperm with normal morphology that were TUNEL negative with fertilization in sibling oocytes, this correlation was not observed in the HOST group. Because sibling oocytes were used, the reasons for the absence of correlation between fertilization rate with percentage of normal morphology that were TUNEL negative in the HOST group could be attributed to the sperm selection procedure. DNA fragmentation in sperm could result in failure to mature, particularly incomplete chromatin packaging that caused sperm to be more prone to DNA fragmentation induced by ROS or other possible sources (23- 27). Therefore, selection of healthy sperm in this procedure might account for improved embryo quality. These results have agreed with the previous study that evaluated the potential for HOST to select healthy sperm in terms of intact chromatin and apoptosis.

Based on the results of previous and current studies, sperm selection based on HOST patterns has been shown to improve sperm quality including chromatin integrity, a low level of apoptosis, and sperm morphology (8). The significantly improved implantation and chemical pregnancy rates, and insignificantly improved clinical pregnancy rate were most likely to be due to the quality of sperm selected by the HOST procedure. Of note, previous literature on IVF couples has also observed significant correlations between the percentage of HOST positive sperm with fertilization, implantation and pregnancy rates (28).

 Although the basis of these observations, according to the literature, have been mainly attributed to sperm motility and viability, today we know these observations could be attributed to sperm functionality or quality. Therefore the HOST pattern selects sperm based on functionality rather than sperm viability. In our previous study we have shown that the percentage of d-sperm or d-HOST pattern sperm had a significant positive correlation with sperm concentration and a negative correlation with sperm DNA fragmentation. A retrospective look at our previous data has revealed that with a higher percentage of HOST positive sperm in a sample, there would be a lower percentage of g-pattern sperm with higher DNA fragmentation. By contrast, in this type of sample, the percentage of d-pattern sperm which shows lower rates of DNA fragmentation is higher (8).

## Conclusion

By taking into consideration the limitation of this study, which was patient preference, therefore the results of this preliminary study and previous study suggested that selection of sperm according to the HOST pattern might potentially improve ICSI outcomes. Despite the improvement in this study and due to the limitations (blinded and randomization) and shortcomings (slightly higher female age in the control group and longer duration of infertility), we have proposed that additional clinical trials are necessary to verify these outcomes.
